# Supplementary material for: Predictive value of somatic and functional variables for cognitive deterioration for early-stage patients with Alzheimer’s Disease: Evidence from a prospective registry on dementia
Source: PLoS One. 2024 Aug 14;19(8):e0307111. doi: 10.1371/journal.pone.0307111 (PMC11324136; doi:10.1371/journal.pone.0307111)

**Supporting information: Manuscript ID PONE-D-23-41598**

Title of manuscript: **Predictive value of somatic and functional variables for cognitive deterioration for early-stage patients with Alzheimer’s Disease: Evidence from a prospective registry on dementia**

**S1 Fig.** **Linear development of patient-related and caregiver variables across time (see also Table 4 of the main text).**


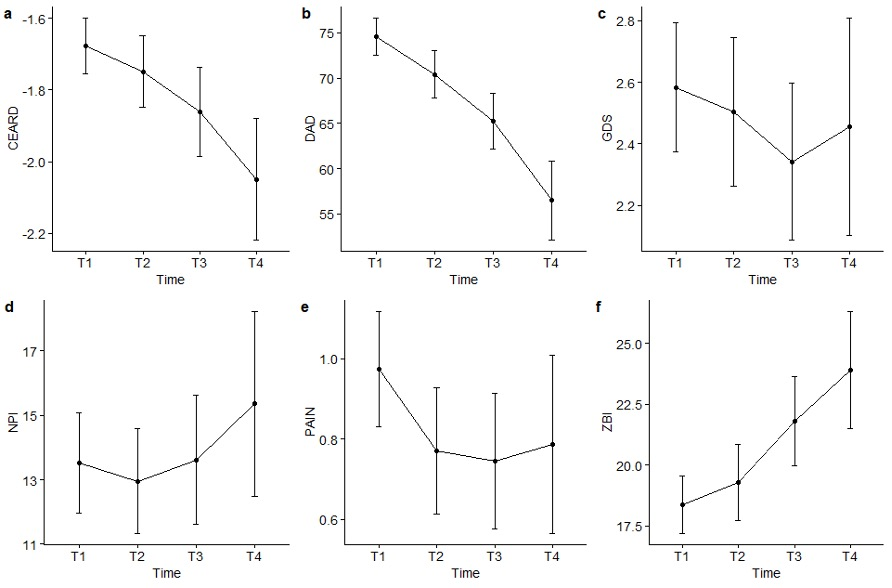


*Notes/Labeling of the y-axes are as follows:* CERAD: mean z-scores of the CERAD subscales (see Defrancesco et al. [21], less negative scores indicating better cognitive performance); DAD: percentage of possible ADLs (lower scores indicating higher functional dependency, max. 100); GDS: total raw score of depressive symptomatology (higher scores indicating more depressive symptoms, max. 15); NPI: total raw score indicating the number of neuropsychiatric symptoms (higher scores indicating more symptoms, max. 144); PAIN: total raw score of activities the patient is no longer able to perform due to pain (higher scores indicating more pain, max. 4); ZBI: total raw score indicating caregiver load (higher scores indicating higher load, max. 88).

*Abbreviations:* CERAD = Consortium to Establish a Registry for Alzheimer’s Disease; DAD = Disability Assessment for Dementia (indexing activities of daily living/ADLs); GDS = Geriatric Depression Scale; PAIN = Pain scale focusing on pain during activity; NPI = Neuropsychiatric Inventory; ZBI = Zarit Burden Inventory.

Reference:

[21] Defrancesco M, Marksteiner J, Kemmler G, Dal-Bianco P, Ransmayr G, Benke T, et al. Specific neuropsychiatric symptoms are associated with faster progression in Alzheimer's disease: Results of the prospective dementia registry (PRODEM-Austria). J Alzheimers Dis. 2020;73:125-33. doi: 10.3233/JAD-190662.

**S2 Fig** **A-E. Scatter plots showing the correlations (R) between the cognitive outcome measure CERAD and the functional patient and caregiver variables (i.e., DAD/activities of daily living, GDS/depressive symptomatology, PAIN/subjective pain, NPI/neuropsychiatric symptoms, ZBI/caregiver load) for each assessment point separately (i.e., T1, T2, T3, T4; also see Table 5 in the main text).**

***S2 Fig A.* Correlations between DAD and CERAD.**


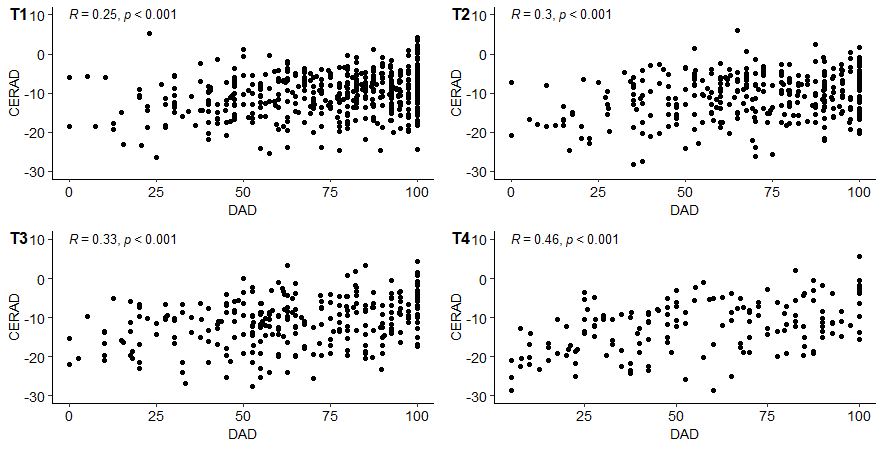


***S2 Fig B. Correlations between GDS and CERAD.***

*
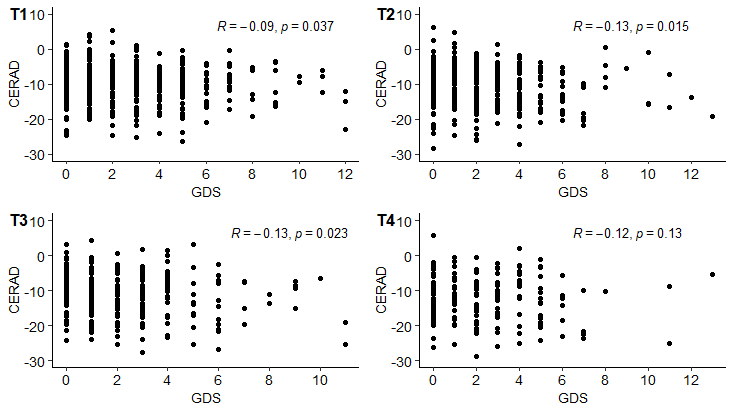
*

***S2 Fig C. Correlations between PAIN and CERAD.***

*
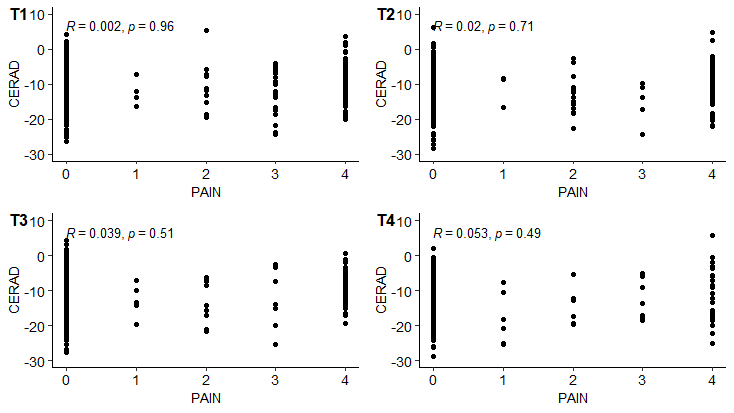
*

***S2 Fig D. Correlations between NPI and CERAD.***

*
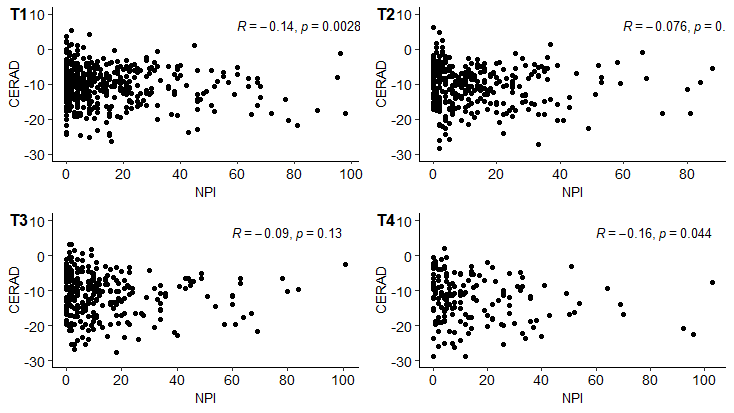
*

***S2 Fig E. Correlations between ZBI and CERAD.***

***
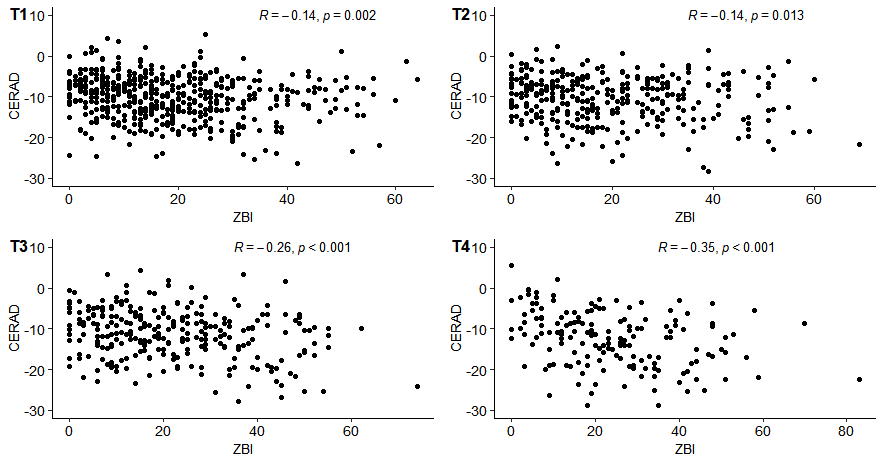
***

**S3 Fig.** **Scale-location graph depicting the squared standardized residuals against the fitted values of our regression model (see Table 6 in the main text).**


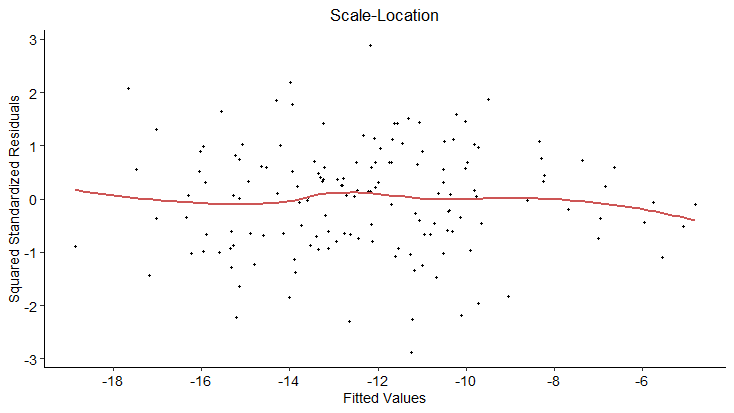


**S4 Fig** **A-D.** **Dot graphs with regression lines depicting the relationship between each significant predictor in our final regression model and the outcome (see Table 6 in the main text)**.


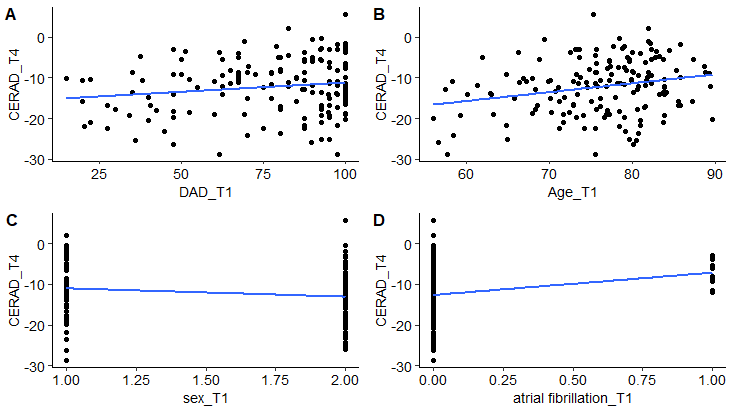

Supplement: S1 File — (DOCX) [file pone.0307111.s001.docx]
