# Supplementary material for: Predictive value of somatic and functional variables for cognitive deterioration for early-stage patients with Alzheimer’s Disease: Evidence from a prospective registry on dementia
Source: PLoS One. 2024 Aug 14;19(8):e0307111. doi: 10.1371/journal.pone.0307111 (PMC11324136; doi:10.1371/journal.pone.0307111)
Supplement: Attachment — Submitted filename: Reviewer feedback.docx [file pone.0307111.s002.docx]

Thank you for the opportunity to review this manuscript. The article was written well and covered a range of literature pertinent to the topic. The findings were also highly interesting and worthwhile for the academic community. I only have a few comments for the authors to consider.

Abstract

Lines 41-42 show an incomplete sentence.

Lines47-50 show repetition in reporting of results.

Introduction

Lines 107 and 109 – two acronyms are used here without explaining their definitions.

Method

Consent – can you clarify the consenting procedures used here? Were there any special considerations/accessibility requirements you had to meet for individuals diagnosed with cognitive decline (particularly as their dementia progressed)?

Data acquisition indicates that family caregivers were involved in the study but the participant eligibility criteria states ‘informal caregivers living with the patient’. Can you clarify this please?

Drop out analysis – could you quantify and report on the reasons for drop out instead of referring to other papers/studies?

General comments

‘Caregiver burden’ is increasingly being recognised as negative towards caregivers. Please consider more person-centred language such as ‘caregiving effects/challenges/load/impact’ as recommended in dementia language guidelines e.g., <https://alzheimer.ca/sites/default/files/documents/Person-centred-language-guidelines_Alzheimer-Society.pdf>
