# Supplementary material for: Predictive value of somatic and functional variables for cognitive deterioration for early-stage patients with Alzheimer’s Disease: Evidence from a prospective registry on dementia
Source: PLoS One. 2024 Aug 14;19(8):e0307111. doi: 10.1371/journal.pone.0307111 (PMC11324136; doi:10.1371/journal.pone.0307111)
Supplement: Attachment — Submitted filename: Response letter_R1_06June2024_final.docx [file pone.0307111.s003.docx]

**Response letter: Manuscript ID PONE-D-23-41598**

**Title:** Predictive value of somatic and functional variables for cognitive deterioration for early-stage patients with Alzheimer’s Disease: Evidence from a prospective registry on dementia.

Dear Editor (dear Dr. Fernandes), dear Reviewers,

thank you very much for inviting us to revise our manuscript. We are pleased that you found our work interesting, and we would like to thank the Reviewers for their constructive and helpful comments. Upon revising the manuscript, we took great care to closely follow all comments raised by you and the Reviewers.

To facilitate the review process, we fully quoted the Reviewers’ comments before the respective reply. Original comments are *italicized* while our replies to these comments are in plain text and preceded by the word Response. Moreover, changes to the main text are indicated in blue font. Please note that references to page numbers and lines refer to the revised manuscript without track changes.

**Editor/Reviewer comments:**

*Dear Dr. Kaufmann,

Thank you for submitting your manuscript to PLOS ONE. After careful consideration, we feel that it has merit but does not fully meet PLOS ONE’s publication criteria as it currently stands. Therefore, we invite you to submit a revised version of the manuscript that addresses the points raised during the review process.

==============================*

*Thank you for submitting your valuable work.*

*The reviews, which are insightful and interesting, pointed to some interesting points. The authors will notice the reviewers found merits in your study, but also raised important concerns.*

*By my own reading, particularly regarding its soundness, stats analysis, and the utilisation of graphs.*

*Also I have some additional suggestions. While these may appear lengthy, the intention is to align more closely with the recommendations while maintaining a high standard of scientific communication. As follows:*

*1) Please double check grammar throughout the text (e.g. there is incorrect comma usage, but what can be refined is avoiding jargon or overly complex sentences that may confuse readers unfamiliar with the topic);*

Response: Done. Upon revising our manuscript, we took great care to also correct the linguistic shortcomings.

*2) Double check the references accordingly to the Journal’s standards (e.g. ref 42 is missing '10.1037//0882-7974.2.3.225', months are not required but check it please, and only the first word of the title and proper nouns are capitalised);*

Response: Done. Apologies for our failure to fully adhere to the author guidelines.

*3) The study encountered the challenge of a high rate over the two-year period, decreasing from 500 participants at baseline to 169 at the final follow-up. While common in longitudinal studies, such attrition can bias the results and affect the study's power to detect changes over time.*

*To mitigate this, the authors can indicate that following studies should implement measures to reduce participant -. This could involve maintaining more regular communication with participants, offering flexible scheduling options to accommodate their needs, and providing reinforcement for their involvement. Additionally, employing advanced statistical methods such as multiple imputation (i.e. if properly used) to handle missing data could aid in mitigating the biases introduced by participant attrition (Sterne et al., 2009). These steps not only demonstrate a commitment to maintaining participant engagement but also enhance the overall robustness and of the findings;*

Response: Thank you for the careful reading of our manuscript. We agree that high drop-out rates need to be carefully checked to ensure reliable and empirically sound data interpretation. Regarding your suggestion to circumvent the high drop-out rate by conducting multiple imputations, we wish to point out that the missing data from the drop-outs are most likely not missing at random (MNAR) but are likely correlated with multiple patient and caregiver parameters (as depicted in our elaboration on the observed reasons for study drop-out, see below). Upon acknowledging previous findings revealing that test results can be easily biased by imputing large amounts of MNAR data (i.e., data that are not missing at random; Van Buuren, 2018), we decided against the multiple imputation of our drop-out data and thus, to avoid biasing our results.

Please note that Reviewer #1 asked for more detailed information on reasons for study drop-out, which we are happy to provide (see end of p. 16, continuing to p. 17 of the response letter).

Reference:

[65] Van Buuren S. *Flexible imputation of missing data*. 2^nd^ edition; Chapman & Hall CRC Press; 2018.

*4) The study's findings from a specific cohort in Austria, may not be generalisable to broader populations due to cultural, genetic, and healthcare system differences. Expanding the research to broaden the research scope to encompass diverse populations across several countries would refine the external validity. This expansion would facilitate a more comprehensive understanding of Alzheimer's Disease (AD).*

*- Furthermore, including people from different educational and economic backgrounds is important. This helps us understand how AD affects various groups in society. By making research more inclusive, we can learn more about its characteristics. If possible, elaborate on past findings;*

Response: We fully agree with the Editor that our findings from an Austrian population are not readily generalizable to individuals from other ethnic backgrounds. However, as this was a prospective study (conducted by the Austrian dementia consortium) we are not able to add any participants or to retrospectively change the study protocol. Accordingly, upon revising our manuscript, we inserted a section on the “Limitations” at the end of the discussion (see pp. 31-32) in which we include this shortcoming as well as other shortcomings mentioned below.

*5) For instance, the study outlines the predictive value of demographic, somatic, and functional variables but may not sufficiently address the potential interaction between these factors. AD progression is influenced by a complex interplay of genetic, environmental, and lifestyle factors, and the additive or multiplicative effects of these variables could provide more insight into the disease’s progression. Please consider employing advanced stats such as structural equation to explore these interactions comprehensively (Livingston et al., 2020);*

Response: We agree that potential interaction effects of our predictor variables need to be considered upon interpreting the findings of our regression analysis. Please note that already in the original paper we tested our data for potential interaction effects – which we did not find – and subsequently, wrote as follows (see p. 16, lines 339-340 of the original ms.): “The final model was checked for collinearity and potential interactions between these predictors.”

Upon following the Editor’s request, we reran our regression model by including all possible interactions between the predictor variables. Importantly, none of the interactions were statistically significant. The latter finding leads us to conclude that we did not miss any significant and/or clinically relevant interactions between the independent variables used in the regression model. Consequently, and upon following the Editor’s request, we elaborated our original statement as follows (see p. 17, lines 374-375 of the revised ms): “Importantly, our findings from stepwise regression analysis seem to be robust and not biased by any potential interaction effects.”

Furthermore, we aimed to run a more complex analysis such as a Structural Equation Model (SEM) on the data from the get-go to explain the longitudinal progression of AD. However, we abandoned this approach because for two main reasons: Firstly, upon employing SEM we would hypothesize causal relationships between the variables of interest (which is not the case in our exploratory and complex study design targeted at (i) examining correlational strengths and (ii) identifying potential predictor variables). Secondly, we could not find a model that reached a sufficient fit (RMSEA>0.08 and CFI<0.9). This also led to contradictory model estimates which we believe would only confuse the readers instead of contributing to the scope of this study.

*6) I'd highly suggest authors to evaluate the model and predictive models, you can use cross-validation (e.g. train and test) or SVM as support for regression analyses. The 'MASS' package is excellent and user-friendly for various regression approaches. For SVM, you can try 'e1071' package. Additionally, for cross-validation, the 'rsample' package could be useful as it allows you to explore different approaches. These won't take more than few minutes.*

*- If the authors want an even more less time-consuming, you try JASP software;*

Response: Upon following your suggestion and supporting the linear regression analysis, we ran a support vector machine regression (SVMR) by using the ‘e1071’ package (and in particular the integrated ‘tune()’ function for optimization). For both regression models, the same variables and study participants were used. Upon using 10-fold validation, the best parameters for our model were an epsilon of 0.6 and a cost of 1.0. Upon comparing both models by the root mean square error (RMSE), results disclosed similar model fits (RMSE_lin. Reg._ = 5.99, RMSE_SVMR_ = 5.76). Thus, the findings from the SVMR corroborate the reported findings of the original linear regression model (please see the Fig below for a direct comparison of the SVMR and our linear regression model as regards the predictive value of the two metric independent variables DAD (indexing activities of daily living) and age at T1 on the cognitive outcome variable (CERAD) at T4.

Please note that for better visualization of the raw data in graphs depicting dichotomous variables (i.e., Fig parts C and D, showing sex: male/female, and atrial fibrillation: yes/no), we used the jittering function. In addition, for the sake of completeness (and to facilitate the review process), we also provide the same graphs (Fig C and D) without the jittering function.

Finally, we wish to point out that our paper is a clinical one and the interested readers most likely are clinicians who are not familiar with sophisticated statistical methods such as SVMR. Hence, we decided not to present this comparison in the revised paper (as the inclusion of the SVMR results would also require a detailed methodological description of SVMR and their usefulness in clinical trials).

Fig (parts A-D) with jittering function:

Fig parts C and D without jittering function:

*- With multiple predictors and outcomes, there's a risk of false-positives. There is the lack of specification of (whether) corrections for multiple testing, such as the Bonferroni correction or FDR. Again, JASP is a very 'catchy' for this;*

*- Considering this, please also emphasise for other researchers that these adjustments to ensure the robustness of findings (Benjamini & Hochberg, 1995). These approaches could provide insights into the underlying mechanisms driving cognitive deterioration in AD;*

Response: Upon following the Editor’s advice, we performed alpha corrections on Table 3 (drop-out analysis) and Table 5 (correlation analysis between functional variables).

With respect to the correlation analysis reported in Table 3, group differences were not significant after applying Bonferroni corrections (*p_min_* = 0.121). Without alpha correction, group differences were significant regarding caregiver burden (ZBI) and two somatic patient variables (i.e., hypercholesterolemia and diastolic blood pressure). Because we aimed to be as sensitive as possible to potential group differences between patients who remained in the study and those who dropped out, we abstained from reporting the alpha-corrected values in Table 3 in the first draft of our manuscript (also see Defrancesco et al., 2020). However, upon following the Editor’s request, we now add the information on alpha-corrected group differences in the Table’s legend (see p. 13, lines 262-264 of the revised ms.) as an amendment of the Note concerning the significance level: “significant results at p < 0.05* (please note that group differences were not anymore statistically significant after applying Bonferroni alpha-corrections, *p_min_* = 0.121)”.

As regards Table 5 (aiming at examining correlation strengths between the functional variables separately reported for the four time points) we wish to emphasize that these correlations are exploratory in nature and thus, aimed at elucidating the complex structure of our data set. Therefore, we didn’t apply an alpha correction to the correlation tables in the first version of our manuscript. However, upon revising our manuscript and for the sake of greater transparency, we adjusted the correlation tables to also provide information about the correlations which remained significant after applying an alpha correction (by inserting a superscript ^§^ to all significant correlations marked with a superscript *). In addition, we now add the following statement to the legend of Table 5 (see “Notes”, p. 21, lines 458-459 of the revised ms.): “^$^significant correlations after Bonferroni alpha correction”.

Reference:

[21] Defrancesco M, Marksteiner J, Kemmler G, Dal-Bianco P, Ransmayr G, Benke T, et al. Specific neuropsychiatric symptoms are associated with faster progression in Alzheimer's disease: Results of the prospective dementia registry (PRODEM-Austria). J Alzheimers Dis. 2020;73:125-33. doi: 10.3233/JAD-190662.

*7) The careful selection of covariates in regression models is essential for accurately estimating effects. However, the study lacks clarification regarding the reasoning behind choosing specific covariates over others. It is imperative to ensure a clear and theory-driven selection of covariates, supported by a pre-analysis plan. This approach helps avoid data dredging and promotes reproducibility (c.f. 10.1097/01.ede.0000056325.26393.17). To maintain the rigour of the findings, it is essential to employ methods such as multiple or sensitivity analyses to assess the impact of missing data on the study's conclusions (c.f. 10.1037/1082-989X.7.2.147; 10.1002/9781119013563);*

Response: We fully agree that the selection of covariates is essential for a meaningful interpretation of the results.

Please note that in our regression analysis (see Table 6, p. 22 of the revised ms) we are testing quite many variables by including them in the stepwise regression analysis. Specifically, we aimed at identifying whether and which patient variables at T1 might contribute significantly to variance explanation of the cognitive outcome variable CERAD at T4. Over and beyond the hypothesized predictors variables (i.e., functional and somatic patient variables as well as the demographic variables age and sex) we also included ‘education’ and ‘time since first diagnosis’ as potential covariates. Since the latter variables (i.e., ‘education’ and ‘time since first diagnosis’) were not selected for the final regression, we conclude that these potential covariates do not contribute significantly to variance explanation of the cognitive outcome variable CERAD at T4. Upon following the request of the Editor, we explicitly checked whether the two potential covariates would critically affect the final regression model. The latter results disclosed that both potential covariates (i.e., ‘education’ and ‘time since first diagnosis’) were statistically not significant (*p_min_* = 0.293) and, moreover, did not change the model fit.

To facilitate the review process, we provide the regression procedure in detail here (and accordingly, elaborate this issue in the section “Statistical methods”, see pp. 16-17 of the revised ms). The stepwise regression was run using the ‘caret’ package. Initially, all previously described and predefined independent variables at T1 (i.e., functional and somatic patient variables as well as demographic variables) were entered to the stepwise regression. The cognitive outcome measure (CERAD total score at T4) was defined as dependent variable. Predictors were sequentially added and removed to find the set of predictors that are best suited for variance explanation. Moreover, 5-fold cross-validation was used to ensure that the result from the stepwise regression would be unbiased. Finally, the regression model with the best fit to our data (i.e., the lowest ‘root mean square error of approximation’/RMSEA) was then used for the following analyses and described in Table 6.

With respect to the Editor’s remark on performing multiple imputations to assess the impact of missing data we would like to refer to our response to comment #3).

*8) While caregiver reports are a practical method in AD research, enhancing them with objective measures or technology-assisted assessments could yield a more precise and holistic insight (c.f. 10.1177/0733464814543965) into the functional abilities and neuropsychiatric conditions of patients (c.f. 10.1093/geront/gnw250). For example, integrating wearable technology for continuous monitoring of physical activity could furnish objective data regarding activities of daily living (ADLs)- that is just an example;*

Response: While we agree with the Editor that technology-assisted assessments are likely to yield more precise and objective insights than caregiver reports (that are subjective in nature), the study design of the present prospective study did not incorporate technology-assisted assessments. We are grateful for the reference suggestions. However, we could not cite the first paper (i.e., the doi does not exist) and the paper belonging to the second reference suggestion (see below) is not targeted at caregivers and/or patients with dementia, and moreover, also used questionnaires rather than technology-assisted or other objective measures. Upon following the Editor’s request, we now include a statement (together with relevant references) on the usefulness of technology-assisted assessments in future research endeavors in the limitation section (see p. 32, lines 716-720). We now write as follows: “Moreover, some of the functional variables rest on patient and caregiver reports (e.g., pain and neuropsychiatric symptoms, respectively) who are subjective in nature and moreover, are characterized by low inter-rater reliability. To obtain objective and precise assessment results, technology-assisted assessment tools seem to be a promising avenue for future research endeavors [66-67].

References:

[66] Maresova P, Tomsone S, Lameski P, Madureira J, Mendes A, Zdravevski E, et al. Technological Solutions for Older People with Alzheimer's Disease: Review. Curr Alzheimer Res. 2018;15:975-83. doi: 10.2174/1567205015666180427124547.

[67] Palmdorf S, Stark AL, Nadolny S, Eliaß G, Karlheim C, Kreisel SH, et al. Technology-Assisted Home Care for People With Dementia and Their Relatives: Scoping Review. JMIR Aging. 2021;4:e25307. doi: 10.2196/25307.

*9) The authors might suggest causal relationships between the predictor variables (e.g. such as atrial fibrillation and ADLs) and cognitive deterioration, even though there may not be convincing evidence of causality.*

*- It's important to interpret these implications with caution, particularly in observational studies where other factors could be influencing the outcome;*

Response: Though we – alike the Editor – believe that our findings are sufficiently robust to warrant an interpretation suggesting causal relationships between predictor variables and cognitive outcome variable, we prefer to interpret our findings more cautiously (especially upon acknowledging the characteristics and potential limitations of our study, such as the fact that our study was observational in nature and – as virtually all clinical prospective studies – characterized by a rather high drop-out rate as well as a questionable generalizability to other populations because the study was national and confined to include patients recruited in Austria).

*10) The Discussion section might generalise the study's findings excessively, overlooking the limitations posed by the methodology, sample demographics, or data analysis.*

*- For example, suggesting that the identified predictors are universally applicable to all early-stage AD patients without acknowledging the cultural, genetic, and healthcare system diversity across different populations could restrict the relevance of the findings.*

*- However, by implementing the suggested techniques or approaches to enhance stats methods and refine findings, it's possible to explore these assertions without overextending the conclusions;*

Response: We would like to thank the Editor for drawing our attention to the additional statistical approaches targeted at corroborating our findings and proofing their robustness. Upon revising our manuscript, we performed additional alpha corrections to our correlation analyses and moreover, calculated SVMR to test the validity of our linear regression models. Consequently, we believe that the quality of our manuscript did improve substantially. Nonetheless, and as already emphasized in our response to comment #9, we prefer a cautious interpretation of our findings. Accordingly, upon revising our manuscript, we introduce a section on the limitations of the study (see pp. 31-32 of the revised manuscript) and moreover, revisit the respective passage in the discussion. In particular, the limitation section now reads as follows:

“Beyond its strengths (i.e., large prospective cohort study comprising a well-defined sample of patients diagnosed with probable and possible AD, targeted at investigating potential predictors of cognitive decline by including somatic and functional patient variables as well as caregiver variables), the present study also has potential limitations. Importantly, our findings might not be readily generalizable to a broader population as our study participants comprised solely Austrian patients with probable and possible AD. Indeed, it has been claimed previously that studies aiming at identifying potential risk factors for dementia incidence and decline are more prevalent in low- and middle-income countries compared to high-income countries (because the former bear a higher risk of potentially modifiable risk factors for dementia [8, also see 3, 60]).

A further potential limitation of the present study are the rather high drop-out rates (i.e., sample size decreasing from initially n=500 participating patients to n=169 at the last follow-up visit). Nonetheless, drop-out rates in prospective studies are frequently reported to be considerable [29-31]. A closer look at the reasons for drop-out disclosed that the missing data is not missing at random (MNAR) but is likely correlated with multiple patient and caregiver parameters. Upon acknowledging previous findings revealing that test results can be easily biased by imputing large amounts of MNAR data (i.e., data that are not missing at random [63]), we decided against the multiple imputation of our (drop-out) data and thus, to avoid biasing our results.

Moreover, some of the functional variables rest on patient and caregiver reports (e.g., pain and neuropsychiatric symptoms, respectively) who are subjective in nature and moreover, are characterized by low inter-rater reliability. To obtain objective and precise assessment results, technology-assisted assessment tools seem to be a promising avenue for future research endeavors [66, 67].

Finally, though our study included quite many potential predictor variables (i.e., somatic and functional patient variables) that were tested for variance explanation of the cognitive outcome variable (indexed by the CERAD), these variables were not comprehensive. Such as, we were not able to include lifestyle factors such as physical activity, nicotine and alcohol use as potential predictors to our study, although it has been argued that these factors should be considered as modifiable risk factors for dementia [8, 9]. Though we did collect patients’ nicotine and alcohol use (but not so physical activity) in our prospective multi-center study, these data were very patchy and thus, could not be introduced to our analyses.”

*11) Also consider tidying the Tables and transferring some to the Sup. Material;*

Response: We are happy to follow the Editor’s suggestion to keep the results section clear by placing the additionally requested Figures in the Supporting Information (e.g., requested additional scale-location graphs as well as classic dot graphs with regression lines, see comments below). In addition, we replaced Appendix A (providing the means and standard deviations of total scores obtained for each functional measure used across the longitudinal assessment) by providing the respective graphs in the Supporting Information (see data in S1 Fig below)

*12) The utilisation of HQ-graphs in the Results section is important. The authors have remarkable and interesting data that could greatly benefit from exploration and visualisation for researchers and readers alike.*

*- For regression analyses (Table 4), employing classic dot graphs with regression lines and scale-location graphs would be particularly insightful. Additionally, incorporating residual plots, especially for the predictors, such as partial regression graphs, could enhance understanding. These graph types can be found in packages like 'MASS' and 'ggfortify', extending beyond the capabilities of classic ggplot;*

Response: Thank you for the positive evaluation of the quality of our study and findings. Upon following the Editor’s request, we now also provide a scale-location graph (see data in S3 Fig below) as well as classic dot graphs with regression lines (see data in S4 Fig A-D below). The dot graphs are shown for the four significant predictors identified by our regression analysis (testing the potential explanatory values of patient and caregiver variables collected at T1 to predict the cognitive outcome measure CERAD at T4).

As our paper is targeted at readers with a clinical background, we would prefer to present these tables in the ESM only. However, if you wish us to transfer these graphs to the main text, we will be happy to do so.

**S3 Fig.** **Scale-location graph depicting the squared standardized residuals against the fitted values of our regression model (see Table 6 in the main text).**

**S4 Fig** **A-D.** **Dot graphs with regression lines depicting the relationship between each significant predictor in our final regression model and the outcome (see Table 6 in the main text)**.

*- For the correlation analysis (Table 5), the authors could either describe the correlations in plain text within the manuscript or utilise scatterplots with correlation coefficients to visually represent the relationships between variables;*

Response: Though we agree that Figures are well suited to visualize results, for economic reasons and moreover, to avoid any redundancies, we chose to report the findings of our complex correlation analyses in the format of a Table (i.e., Table 5). Please note that in addition to reporting our correlation findings in Table 5, we discuss the significant and clinically relevant findings already in the results section (see pp. 19-21 of the revised manuscript). Nonetheless, upon following your request, we now provide the requested scatterplots in the Supporting Information (see data in S2 Fig A-E). Due to the high number of correlations reported in Table 5, we chose to focus on the correlations with our cognitive outcome variable CERAD (shown separately for each of the four time points).

**S2 Fig** **A-E. Scatter plots showing the correlations (R) between the cognitive outcome measure CERAD and the functional patient and caregiver variables (i.e., DAD/activities of daily living, GDS/depressive symptomatology, PAIN/subjective pain, NPI/neuropsychiatric symptoms, ZBI/caregiver load) for each assessment point separately (i.e., T1, T2, T3, T4, see Table 5).**

***S2 Fig A. Correlations between DAD and CERAD.***

***S2 Fig B. Correlations between GDS and CERAD.***

***S2 Fig C. Correlations between PAIN and CERAD.***

***S2 Fig D. Correlations between NPI and CERAD.***

***S2 Fig E. Correlations between ZBI and CERAD.***

*- I'd highly suggest that the authors examine the autocorrelation function to assess the presence of seasonality. Additionally, for the LMM, it would be beneficial for the authors to present the main findings in plain text and complement them with representations over time. Polar graphs or line graphs depicting differences across time points could effectively illustrate these findings;*

Response: We agree that autocorrelations need to be examined. Please note that already in the original version of our manuscript, the calculation of our LMM (**Table 7**) included testing for autocorrelations for t-1. However, we are happy to follow the Editor’s advice and provide the respective Figure below. By doing so, we employed an ACF-plot to visualize potential autocorrelations of the residuals. As can be seen from the Figure, autocorrelations are not present in our LMM (as depicted in **Table 7**). Thus, we are confident that seasonal effects did not influence the measurements and therefore the results of our analysis.

**Fig.** **ACF plot showing no substantial autocorrelation in the Linear Mixed Model (see Table 7 in the main text).**

Moreover, as requested, we now provide graphs to visualize the development of the functional variables over time (i.e., indicating disease progression). Please note that in the original manuscript, we reported the exact data on functional developmental trajectories in table format (see former Appendix A). Upon following your comments suggesting (i) to provide more illustrative graphs, and (ii) to tidy the results section by reducing Tables, we deleted Appendix A from the main text and, instead, provide the same information in graph form in the Supporting Information (see data in S1 Fig below).

**S1 Fig.** **Linear development of patient-related and caregiver variables across time (see also Table 4 of the main text).**

*Notes/Labeling of the y-axes are as follows:* CERAD: mean z-scores of the CERAD subscales (see Defrancesco et al. [21], less negative scores indicating better cognitive performance); DAD: percentage of possible ADLs (lower scores indicating higher functional dependency, max. 100); GDS: total raw score of depressive symptomatology (higher scores indicating more depressive symptoms, max. 15); NPI: total raw score indicating the number of neuropsychiatric symptoms (higher scores indicating more symptoms, max. 144); PAIN: total raw score of activities the patient is no longer able to perform due to pain (higher scores indicating more pain, max. 4); ZBI: total raw score indicating caregiver load (higher scores indicating higher load, max. 88).

*Abbreviations:* CERAD = Consortium to Establish a Registry for Alzheimer’s Disease; DAD = Disability Assessment for Dementia (indexing activities of daily living/ADLs); GDS = Geriatric Depression Scale; PAIN = Pain scale focusing on pain during activity; NPI = Neuropsychiatric Inventory; ZBI = Zarit Burden Inventory.

Reference:

[21] Defrancesco M, Marksteiner J, Kemmler G, Dal-Bianco P, Ransmayr G, Benke T, et al. Specific neuropsychiatric symptoms are associated with faster progression in Alzheimer's disease: Results of the prospective dementia registry (PRODEM-Austria). J Alzheimers Dis. 2020;73:125-33. doi: 10.3233/JAD-190662.

*------*

*Please bear that these suggestions are intended to enhance the quality and presentation of your findings for researchers and readers. By implementing these recommendations, you can streamline your presentation by reducing the number of Tables and making your data more engaging. Given the potential of your study to attract a broad audience, it's essential to maintain rigorous approaches and ensure clear and engaging presentation, just as you have done with your study design and data collection.*

*Hope the authors find all (or most) of the suggestions useful and hope this helps somehow.*

*Wishing you success with the study*

*==============================*

**Reviewers' comments:**

*Reviewer's Responses to Questions*

***Comments to the Author*** *1. Is the manuscript technically sound, and do the data support the conclusions?

The manuscript must describe a technically sound piece of scientific research with data that supports the conclusions. Experiments must have been conducted rigorously, with appropriate controls, replication, and sample sizes. The conclusions must be drawn appropriately based on the data presented.*

*Reviewer #1: Yes*

*Reviewer #2: Yes*

*Reviewer #3: Yes*

*2. Has the statistical analysis been performed appropriately and rigorously?*

*Reviewer #1: Yes*

*Reviewer #2: Yes*

*Reviewer #3: Yes*

*3. Have the authors made all data underlying the findings in their manuscript fully available?

The* [*PLOS Data policy*](http://www.plosone.org/static/policies.action#sharing) *requires authors to make all data underlying the findings described in their manuscript fully available without restriction, with rare exception (please refer to the Data Availability Statement in the manuscript PDF file). The data should be provided as part of the manuscript or its supporting information, or deposited to a public repository. For example, in addition to summary statistics, the data points behind means, medians and variance measures should be available. If there are restrictions on publicly sharing data—e.g. participant privacy or use of data from a third party—those must be specified.*

*Reviewer #1: Yes*

*Reviewer #2: Yes*

*Reviewer #3: Yes*

*4. Is the manuscript presented in an intelligible fashion and written in standard English?

PLOS ONE does not copyedit accepted manuscripts, so the language in submitted articles must be clear, correct, and unambiguous. Any typographical or grammatical errors should be corrected at revision, so please note any specific errors here.*

*Reviewer #1: Yes*

*Reviewer #2: Yes*

*Reviewer #3: Yes*

***5. Review Comments to the Author***
 *Please use the space provided to explain your answers to the questions above. You may also include additional comments for the author, including concerns about dual publication, research ethics, or publication ethics. (Please upload your review as an attachment if it exceeds 20,000 characters)*

***Reviewer #1:***

*Thank you for the opportunity to review this manuscript. The article was written well and covered a range of literature pertinent to the topic. The findings were also highly interesting and worthwhile for the academic community. I only have a few comments for the authors to consider.

Abstract
Lines 41-42 show an incomplete sentence.*

Response: Done

*Lines47-50 show repetition in reporting of results.*

Response: Apologies for the unclear presentation of our findings. Please note that the reported results are derived from two separate analyses (as already stated explicitly in the first version of our manuscript, please see first paragraph under the subtitle ‘Predictive variables for cognitive deterioration’ (pp. 21-23 of the revised ms). While in the first analysis, we report findings from a stepwise regression analysis targeted at investigating the predictive value of variables collected at T1 on the outcome variable (CERAD) at T4, the results of the second analysis are based on a linear mixed model analysis that included the variables of all four assessment points (i.e., T1, T2, T3 and T4) for predicting the outcome variable at T4*.* Upon revising our manuscript, we made the differences between the two regression analyses clearer (both in the ‘Abstract’ as well as in the section ‘Statistical methods’).

*Introduction
Lines 107 and 109 – two acronyms are used here without explaining their definitions.*

Response: Apologies for our failure to explain the acronyms (i.e., MMSE, ADLs). Upon revising the ms., we inserted the full names of these acronyms (i.e., Mini-Mental-Status-Examination and Activities of Daily Living, respectively).

*Method
Consent – can you clarify the consenting procedures used here? Were there any special considerations/accessibility requirements you had to meet for individuals diagnosed with cognitive decline (particularly as their dementia progressed)?*

Response: We are grateful for this valuable comment. Please note that already in the first version of our manuscript, we explicitly state that patients unable to provide informed consent were excluded from study participation. Upon revising our manuscript, we now elaborate this sentence and are writing as follows (cursive letters in black indicating the original statement; see p. 9, lines 198-202): “*Excluded were patients with reduced capacity to consent and/or who were unable to sign an informed consent or if co-morbidities were likely to preclude study termination*. Importantly, reduced capacity to consent was an exclusion criterion at all time points (i.e., T1 to T4). In our sample, no single patient had to be excluded at the follow-up visits because of severe cognitive deterioration yielding reduced capacity to consent and/or difficulties to understand the medical and neuropsychological procedures associated with the follow-up visits.”

*Data acquisition indicates that family caregivers were involved in the study but the participant eligibility criteria states ‘informal caregivers living with the patient’. Can you clarify this please?*

Response: Apologies for the inconsistent terminology. In the revised paper, and upon citing the literature, we explicitly explain that in the cited studies, the term ‘informal caregivers’ comprise family members, neighbours and friends (see p. 6, lines 115-117 of the revised ms). Moreover, in the revised manuscript, we are consistently using the term ‘family caregivers’ instead of ‘informal caregiver’.

*Drop out analysis – could you quantify and report on the reasons for drop out instead of referring to other papers/studies?*

Response: Thank you for the careful reading of our manuscript. In the present study cohort, drop-outs occurred due to the following reasons: Most notably, caregiver stress or illness emerged as significant contributors, resulting in an inability for patients to consistently adhere to follow-up appointments. The latter argumentation is supported by results of the drop-out analysis revealing a significant differences of caregiver load (indexed by ZBI/Zarit Burden Inventory, higher values reflecting higher caregiver load) between caregivers of patients who remained in the study (mean ZBI 16.28/SD 11.78) and caregivers of those who dropped out (mean ZBI 19.39/SD 14.07; *p* = 0.031, see Table 3). Moreover, a substantial proportion of participants, exceeding 50 percent, expressed a perceived decline in the benefit derived from their continued participation as the study progressed. Additionally, approximately 25 percent of study participants faced challenges related to transportation logistics or scheduling conflicts, impeding their continued engagement, and leading to voluntary withdrawal from follow-up visits. Moreover, a minority of participants, accounting for less than ten percent, encountered health complications or comorbidities that compromised their capacity to remain enrolled in the study. Importantly, most of these health issues were discerned to be independent of dementia pathology.

*General comments
‘Caregiver burden’ is increasingly being recognised as negative towards caregivers. Please consider more person-centred language such as ‘caregiving effects/challenges/load/impact’ as recommended in dementia language guidelines e.g., https://alzheimer.ca/sites/default/files/documents/Person-centred-language-guidelines_Alzheimer-Society.pdf*

Response: We would like to thank the reviewer for drawing our attention to the person-centred use of language and for the link to the respective guidelines of the Alzheimer Society. Upon revising our manuscript, we took great care to consistently use person-centred language (i.e., changing the term ‘caregiver burden’ to ‘caregiver load’ or ‘challenges related to the caregiving situation’). However, please note that the instrument used to assess caregiver load in the present study is the so-called ‘Zarit Burden Inventory’ (ZBI). Thus, whenever citing this questionnaire we used the proper name.

***Reviewer #2****:*

*The authors have done a commendable job of presenting a study that assesses biological/somatic and functional variables associated with cognitive decline in early AD. The correlation between caregiver burden and patient-related measures in the early disease stages will be of particular interest to clinicians as they prepare their clinical interview/test battery. Finally, the generation of predictive variables that best explain cognitive decline over time is noteworthy.*

Response: Thank you for the positive overall evaluation of our work!

*The authors can consider fleshing out the discussion subsection ‘Predictive variables for cognitive deterioration’ to address the following:
- Contextualise how the fit of their model compares to others previously published that seek to determine the role of age/sex in contributing to cognitive decline risk. The authors could put forward rationale for the possible modest fit of their model. This comment is not meant to question the value of the current model.*

Response: Thank you for this valuable comment. Upon following the Reviewer’s request, we now discuss potential reasons for our model fits (suggesting medium effect sizes according to Cohen, 1988 as regards both the stepwise regression analysis depicted in Table 6 and the linear mixed model depicted in Table 7) and how our findings relate to the respective relevant literature. In particular, we now write as follows (see pp. 27-28, lines 611-629): “Overall, our model fits (suggesting medium effect sizes according to Cohen [49]) are good, especially upon acknowledging that our findings are quite complex and influenced by quite many factors, some of which we were able to assess, while we could not examine other factors that have been reported to influence cognitive deterioration such as lifestyle factors (physical activity, nicotine and alcohol consumption, cognitive (in)activity etc. [8, 9]. Hence, although we entered quite many potential predictor variables to our stepwise regression model initially, our model might not be complete as we were not able to include all potentially relevant predictor factors for cognitive deterioration. Further potential reasons for our – at first glance - rather modest model fit are the following: (i) our prospective study aimed at investigating disease progression in early-stage dementia over a relatively short period of two years (which is very brief upon considering that dementia per definition is a slowly progressive neurodegenerative disease [1-4, 57]); (ii) the high drop-out rate clearly hampered our model fit; and (iii) some of the measures and tests used were observational and/or based on subjective patient/caregiver reports and thus, possibly less sensitive than objective assessment tools. However, variance explanations of 14 % and 16 % (see Tables 6 and 7, respectively) are quite remarkable upon acknowledging that the functional changes were observed in early stages of a neurodegenerative disease and within a short time span of two years.”

*- Provide a bit more explanation/speculation about why a) education was not a significant factors in their results/model and b) atrial fibrillation but not other somatic factors was a significant predictor of cognitive decline especially when the authors mention “The current scientific discourse on potential somatic risk factors, particularly those associated with cardiovascular health, remains inconclusive (lns 604-606). The authors have provided suitable ref(s) and general statements but a bit more explanation would be beneficial.*

Response: As requested, we now elaborate on the respective part of the discussion (see p. 30, lines 666-674 of the revised manuscript). We now write as follows: “On the other hand, education failed to emerge as a predictor of cognitive functioning or deterioration in our prospective study. Though converging evidence suggests that low education increases the risk of dementia (for the findings of a respective meta-analysis, see 59], our results propose that education seems less important for disease progression if other relevant risk factors are considered simultaneously (for similar findings see [57]). These results align with the idea that, although educational attainment contributes to individual differences across the lifespan, it does not necessarily serve as a protective factor against cognitive decline [59, 60].

In addition, and further below, we elaborate as follows (see pp. 30-31, lines 679-693): “Consistent with our findings, there is accumulating evidence supporting the association between atrial fibrillation and cognitive impairment [61, 62]. This link persists even in early-onset dementia [63]. Furthermore, several studies have shown that atrial fibrillation not only increases the risk of vascular dementia but also the risk of AD. Such as, the findings of a meta-analysis published in 2021 disclose that patients with atrial fibrillation had a 60% higher risk of incident all-cause dementia, a 40% higher risk of incident AD, and a 70% higher risk of incident vascular dementia [64]. Most likely, the relationship is multifactorial in nature, involving inflammatory, hemodynamic and/or genetic components. As noted by Rivard et al. [62], prospective large-scale studies are required to establish the causal effects of this relationship. Filling this gap, our prospective study found that even in the early stages of the disease, and beyond other somatic risk factors, atrial fibrillation should be considered a significant and clinically relevant risk factor for cognitive deterioration and, consequently, AD progression.”

*Minor points:
- Lns41-42: “variables for cognitive deterioration” (unclear sentence structure?)*

Response: Apologies for this flaw. Upon revising our manuscript, we now write as follows: “Though quite many risk factors for disease progression have been identified, there is a lack of prospective studies investigating the interplay and predictive value of a wide variety of patient variables associated with cognitive deterioration (defined as key feature of AD progression).”

*- Ln75.. Section on dementia risk factors could include Livingston papers*

Response: Thank you for drawing our attention to the influential work of Gill Livingston. Upon revising our manuscript, we now include the references [8] and [9], see p. 4, lines 79-80 of the revised manuscript).

[8] Livingston G, Huntley J, Sommerlad A, Ames D, Ballard C, Banerjee S, et al. Dementia prevention, intervention, and care: 2020 report of the Lancet Commission. Lancet. 2020 Aug 8;396(10248):413-446. doi: 10.1016/S0140-6736(20)30367-6

[9] Montero-Odasso M, Ismail Z, Livingston G. One third of dementia cases can be prevented within the next 25 years by tackling risk factors. The case "for" and "against". Alzheimers Res Ther. 2020 Jul 8;12(1):81. doi: 10.1186/s13195-020-00646-x

*- Table 1: caregiver sample size does not appear to add up*

Response: Apologies, but we do not understand the reviewer’s comment. Please note that in Table 1, sample sizes of participating patients and caregivers perfectly match (i.e., all participating patients had a family caregiver who also took part in the study). As we were interested to identify potential patient as well as potential caregiver predictors for cognitive deterioration, only patients who had a family caregiver willing to also participate were included in the present longitudinal study.

***Reviewer #3:***

*This is important topic and the paper is well organized and written. but couples of items should be addressed*

Response: Thank you for the positive overall evaluation of our work!

*1. remove CERAD, this is not the mesh key word*

Response: Done!

*2. The current study seems very confusing and messy with multiple variables. Authors need to work more on the introduction to support 1) why caregiver burden is added to as one of the variables, it is well known from the previous studies, what are the lacking in this caregiver topic.*

Response: Upon following the Reviewer’s suggestions, we now provide a better description of our study variables and procedures in the ‘Statistical methods’ section (see pp. 16-18 of the revised manuscript). Please note that already in the first version of the manuscript, we provide a detailed explanation of our research hypotheses and procedures (see section ‘Study aims’, pp. 6-7 of the revised manuscript) as well as a schematic figure depicting the study variables and predictions (see Fig 1).

Moreover, we elaborate our rationale to include caregiver load as a potential predictor to our regression models (see p. 6, lines 124-130 of the revised ms). We now write as follows: “Clearly, the presence of neuropsychiatric symptoms (that can be differentiated into behavioral, emotional, and psychotic behaviors [21]) and/or higher functional dependency (e.g., increasing need for support to manage daily affairs) is unequivocally associated with an increasing impact on caregivers’ physical and mental health [22, 24-26]. Hence, the examination of the interplay between caregiver load and patient-related measures - especially during early stages of AD - is of utmost interest.“

*3. methods-results: all mixed together with measures, statistical analyses, and results, please write clearly to separate methods (measures and statistical analysis),*

Response: Thank you for this valuable comment. Upon revising our manuscript, we took great care to clearly describe the measures (tests) and variables used and further, we further elaborated the ‘Statistical methods’ section (see pp. 16-18 of the revised ms).

*4. statistical analys: how did authors identify covariates, what covariates were controlled. 4. chi square test: authors need to address multiple comparison such as bonferronni correction. so many variables comparision will have type I error concerns of false positive results.*

Response: As the same comments have been raised by the Editor, we would like to refer the Reviewer to the respective responses. With respect to your concern raised regarding the identification of covariates, please see our response to the Editor’s comment #7.

Regarding your suggestion to employ alpha corrections to adjust for multiple testing, please see our response to the Editor’s comment #6.

Our response to the Editor’s comment #6 (alpha correction):

Response: Thank you for the careful reading of our manuscript. Upon following the Editor’s advice, we performed alpha corrections on Table 3 (drop-out analysis) and Table 5 (correlation analysis between functional variables).

With respect to the correlation analysis reported in Table 3, group differences were not significant after applying Bonferroni corrections (*p_min_* = 0.121), while without alpha correction, group differences were significant regarding two somatic patient variables (i.e., hypercholesterolemia and diastolic blood pressure). Because we aimed to be as sensitive as possible to potential group differences between patients who remained in the study and those who dropped out, we abstained from reporting the alpha-corrected values in Table 3 in the first draft of our manuscript (also see Defrancesco et al., 2020). However, upon following the Editor’s request, we now add the information on alpha-corrected group differences in the Table’s legend. Such as, we now write as follows (as an amendment of the Note concerning the significance level: “significant results at p < 0.05* (please note that group differences were not anymore statistically significant after applying Bonferroni alpha-corrections, *p_min_* = 0.121)”.

As regards Table 5 (aiming at examining correlation strengths between the functional variables separately reported for the four time points) we wish to emphasize that these correlations are exploratory in nature and thus, aimed at elucidating the complex structure of our data set. Therefore, we didn’t apply an alpha correction to the correlation tables in the first version of our manuscript. However, upon revising our manuscript and for the sake of greater transparency, we adjusted the correlation tables to also provide information about the correlations which remained significant after applying an alpha correction (by inserting a superscript § to all significant correlations marked with a superscript *). In addition, we now add the following statement to the legend of Table 5 (see “Notes”, p. 21, line 458-459): “^$^significant correlations after Bonferroni alpha correction”.

Reference:

[21] Defrancesco M, Marksteiner J, Kemmler G, Dal-Bianco P, Ransmayr G, Benke T, et al. Specific neuropsychiatric symptoms are associated with faster progression in Alzheimer's disease: Results of the prospective dementia registry (PRODEM-Austria). J Alzheimers Dis. 2020;73:125-33. doi: 10.3233/JAD-190662.

*Our response to the Editor’s comment #7:*

Response: We fully agree that the selection of covariates is essential for a meaningful interpretation of the results.

Please note that in our regression analysis (see Table 6, p. 22 of the revised manuscript) we are testing quite many variables by including them in the stepwise regression analysis. Specifically, we aimed at identifying whether and which patient variables at T1 might contribute significantly to variance explanation of the cognitive outcome variable CERAD at T4. Over and beyond the hypothesized predictors variables (i.e., functional and somatic patient variables as well as the demographic variables age and sex) we also included ‘education’ and ‘time since first diagnosis’ as potential covariates. Since the latter variables (i.e., ‘education’ and ‘time since first diagnosis’) were not selected for the final regression, we conclude that these potential covariates do not contribute significantly to variance explanation of the cognitive outcome variable CERAD at T4. Upon following the request of the Editor, we explicitly checked whether the two potential covariates would critically affect the final regression model. The latter results disclosed that both potential covariates (i.e., ‘education’ and ‘time since first diagnosis’) were statistically not significant (*p_min_* = 0.293) and moreover, did not change the model fit.

To facilitate the review process, we provide the regression procedure in detail here (and accordingly, elaborate this issue in the section ‘Statistical methods’ in the revised manuscript, see pp. 16-18 of the revised ms). The stepwise regression was run using the ‘caret’ package. Initially, all previously described and predefined independent variables at T1 (i.e., functional and somatic patient variables as well as demographic variables) were entered to the stepwise regression. The cognitive outcome measure (CERAD total score at T4) was defined as dependent variable. Predictors were sequentially added and removed to find the set of predictors that are best suited for variance explanation. Moreover, 5-fold cross-validation was used to ensure that the result from the stepwise regression would be unbiased. Finally, the regression model with the best fit to our data (i.e., the lowest ‘root mean square error of approximation’/ RMSEA) was then used for the following analyses and described in Table 6.

*5. discussion need more work: what are not known (so many published papers already to show the relationships with psych, functional decline, and caregiver burden with AD). and address what parts of this study is adding to the existing results. Also, please address future intervention based on the results, and "so what" with the research findings*

Response: We are grateful for these valuable comments. Upon following the Reviewer’s suggestion, we further elaborate on the novelty of our study findings in the section entitled ‘What is the added value of the latter findings and how do they contribute to a better understanding of AD?’ (see p. 29, lines 643-657 of the revised manuscript). We now write as follows: “Upon acknowledging the complex nature of our research questions, it does not come as a surprise that also our findings are complex and far from being comprehensive. However, our prospective multi-center study is among the first to simultaneously examine patients’ demographic, somatic and functional variables as potential predictor variables for disease deterioration (indexed by the CERAD) in patients with early-stage AD. Furthermore, we aimed at elucidating the developmental trajectories of cognitive and non-cognitive functional variables across time (i.e., four assessment points spanning two years). Our findings are significant as they clearly contribute towards a deeper understanding of AD. Above all, our findings might inform future studies targeted at elucidating the complex interplay of patient and caregiver-related predictor variables (e.g., by providing a starting point for more specific hypotheses, by utilizing objective rather than subjective test measures that -among others- may facilitate cross-national comparisons and thus, enhance the reliability and generalizability of respective research findings).“

Furthermore, we discuss how our findings could inform treatment and intervention of patients with AD (see section ‘Conclusion’, p. 33, lines 744-753 of the revised manuscript). We now write as follows: “Such as, our findings disclosing strong correlations between caregiver load and various patient-related measures strongly suggest that the clinical management of early-stage AD should be targeted at the patient-caregiver dyad (instead of focusing on the patient solely). Moreover, our regression findings disclose that a combination of high age, female sex, atrial fibrillation (above and beyond other somatic risk factors), low ADLs and depressive symptomatology should be considered as significant (and partly modifiable) risk factors [8, 9] for cognitive deterioration. Hence, we propose that a comprehensive treatment for early-stage AD calls for a multidisciplinary approach, including medical, (neuro)psychological and therapeutic professions.“

Altogether, we believe that the quality of our manuscript did benefit from these amendments.
